# Towards an automated approach for understanding problematic gaming

**DOI:** 10.3389/fspor.2024.1407848

**Published:** 2024-08-09

**Authors:** Ana Paula Afonso, Manuel J. Fonseca, Joana Cardoso, Beltrán Vázquez

**Affiliations:** ^1^ LASIGE, Faculdade de Ciências, Universidade de Lisboa, Lisboa, Portugal; ^2^ Department of Social and Behavioural Sciences, University of Maia, Maia, Portugal; ^3^ Center for Psychology at the University of Porto, Porto, Portugal; ^4^ School of Health, Center for Rehabilitation Research, Polytechnic Institute of Porto, Porto, Portugal; ^5^ Escola Superior de Saúde, Instituto Politécnico do Porto, Porto, Portugal

**Keywords:** video games, Internet Gaming Disorder, gamer behavior, telemetry data, emotional states

## Abstract

**Introduction:**

Video games have become increasingly popular worldwide, attracting billions of gamers across diverse demographics. While studies have highlighted their potential benefits, concerns about problematic gaming behaviors have also emerged. Conditions such as Internet Gaming Disorder (IGD) have been recognized by major health organizations, necessitating accurate diagnostic tools. However, existing methods, primarily reliant on self-report questionnaires, face challenges in accuracy and consistency. This paper proposes a novel technological approach to provide gaming behavior indicators, aiming to offer precise insights into gamer behavior and emotion regulation.

**Methods:**

To attain this objective, we investigate quantifiable gaming behavior metrics using automated, unobtrusive, and easily accessible methods. Our approach encompasses the analysis of behavioral telemetry data collected from online gaming platforms and incorporates automated extraction of gamer emotional states from face video recordings during gameplay. To illustrate the metrics and visualizations and demonstrate our method’s application we collected data from two amateur and two professional gamers, all of whom played Counter-Strike2 on PC. Our approach offers objective insights into in-game gamer behavior, helping health professionals in the identification of patterns that may be difficult to discern through traditional assessment methods.

**Results:**

Preliminary assessments of the proposed methodology demonstrate its potential usefulness in providing valuable insights about gaming behavior and emotion regulation. By leveraging automated data collection and visualization analysis techniques, our approach offers a more comprehensive understanding of gamer behavior, which could enhance diagnostic accuracy and inform interventions for individuals at risk of problematic gaming behaviors.

**Conclusion:**

Our findings demonstrate the valuable insights obtainable from a tool that collects telemetry data, emotion regulation metrics, and gaming patterns. This tool, utilizing specific indicators, can support healthcare professionals in diagnosing IGD and tracking therapeutic progress, potentially addressing challenges linked to conventional IGD assessment methods. Furthermore, this initial data can provide therapists with detailed information on each player’s problematic behaviors and gaming habits, enabling the development of personalized treatments tailored to individual needs. Future research endeavors will focus on refining the methodology and extending its application in clinical settings to facilitate more comprehensive diagnostic practices and tailored interventions for individuals at risk of problematic gaming behaviors.

## 1 Introduction

Video games are a major human activity that, in the last decades, have become increasingly more complex, diverse, realistic, and socially interactive, and are played by billions of people across geographical areas, cultures, and demographics (1). A recent report in Europe (2) concludes that: (i) 53% of the population aged 6–64 plays video games; (ii) games are played by more than two-thirds of children and adolescents; and (iii) a substantial number of adults also play games. The average age of gamers is 32 years old, and 74% play at least one hour/week, 17% one hour/month, and 9% once a year, and the average playtime is 8.8 h per week (2).

Over the past years, several studies have been undertaken to analyze the benefits on an individual’s physical, mental, and social well-being (3–5). Some studies suggest that video games enhance cognitive abilities (6), improve socialization, provide stress relief, can have an educational value (7), can help gamers improve problem-solving skills, and can improve hand-eye coordination and fine motor skills (8).

Nevertheless, some problematic gaming behaviors have emerged, and the use of video games can have adverse effects related to depression, aggression, and physical health problems (9–12), pathological and even addictive (13–15). This problematic usage of video games can be associated with some factors. For example, some game genres have been associated with a higher prevalence of addictive behaviors, such as massively multiplayer online role-playing games, first-person shooters, and real-time strategy games/Multiplayer Online Battle Arena (MOBA) (16). On the other hand, various studies have yielded conflicting results regarding the relationship between gaming time and problematic gaming behavior. While some research suggests a link between increased gaming time and addiction [e.g., (17)], other studies have found contradictory evidence [e.g., (18)]. The recent international policy decisions evidence an attempt to resolve the scientists’ uncertainties regarding the consequences of video game use (19, 20).

Due to the emergence of these problematic gaming behaviors, a disorder related to video game addiction emerged in the Diagnostic and Statistical Manual of Mental Disorders (DSM-5), in 2013, in the section of conditions for future study to motivate discussion and research (19). This disorder, named Internet Gaming Disorder (IGD), can be diagnosed using a list of nine diagnostic criteria, requiring evidence of at least five symptoms over a period of at least 12 months. Although the duration may be shortened in cases of severe symptoms that meet all diagnostic requirements (19). The severity of IGD can range from mild to severe, and it can affect people of all ages, although it is more common among younger individuals. Some examples of these criteria are withdrawal symptoms, constant preoccupation with video games, loss of interest in other activities, and lying/deceiving people or health professionals about the time spent playing, among others.

Additionally, the World Health Organization (WHO), five years later, also recognized Gaming Disorder (GD) in the International Classification of Diseases (ICD-11) and classified it as behavioral addiction related to video games (20). GD is characterized by a pattern of continuous or recurrent gaming behavior manifested by three symptoms: lack of control over gaming habits, prioritizing gaming over other interests and activities, and continuing to play games despite negative consequences. This pattern of gaming behavior may result in significant impairment in personal, social, educational, or occupational areas of functioning (20). The severity of IGD can range from mild to severe, can affect people of all ages, ethnicities, educational levels, or geographical distribution. According to Stevens et al. (21), the worldwide prevalence is 3.05%, while in Europe, it ranges from 0.8% to 11.8% (22).

Mental health professionals typically diagnose IGD using criteria outlined in the DSM-5 or the ICD-11, which requires careful assessments by therapists (23), and a range of factors need to be considered, such as severity, duration, and consequences of the gaming behavior, as well as any co-occurring mental health issues and family environment. Currently, to diagnose this disorder, self-report questionnaires are used as part of the assessment process and typically use a combination of clinical interviews, assessment tools, and other sources of information, such as reports from family members or close friends, medical records, and behavioral observations (23). Several self-report questionnaires have been commonly used in research and clinical practice to assess IGD (24), namely, IGDS9-SF (Internet Gaming Disorder Scale–Short-Form) (25), AIGDT-10 (26), video game addiction test (27), and Gaming Addiction Identification Test (GAIT) (28). Some tools, such as IGDS9-SF, are based on criteria like those found in the DSM-5, while others have unique criteria. The scoring and interpretation of the results may differ from one tool to another, which could impact how clinicians or researchers assess the severity of IGD (25) and can provoke significant differences in prevalence rates, diagnostic accuracy, and comparability of research findings across studies (29). Additionally, the self-report approaches have unavoidable limitations, namely, biased recall, denial/defensiveness, and lack of insight (23, 24). For instance, they are inaccurate as subjective responses of the gamer hardly capture the real gamer behaviors, as they may not remember or not mention some relevant information (e.g., year playtime, last week playtime, emotional states). A recent survey (24) underscores the ongoing uncertainty and a lack of consensus within the research community regarding the best practices for screening and assessing IGD. Researchers and clinicians continue to work towards establishing a consensus on the criteria used for screening and diagnosing IGD and develop a comprehensive standard assessment approach that considers the multidimensional nature of gaming behavior and its associated factors (24). Nevertheless, researchers and mental health professionals are also exploring alternatives to traditional assessment methods to improve the accuracy and validity of the diagnosis of IGD and to be used in conjunction with other clinical assessments and observations.

While recent research has suggested a limited influence of the time spent playing video games on overall well-being (30), it remains crucial to obtain precise insights into gamers behavior, emphasizing the characteristics of their gameplay experiences, in-game events, and the specific gamer profiles for which effects may differ (31) and emotion regulation remain crucial for understanding IGD (32, 33).

Nowadays, large volumes of game data are recorded daily through game telemetry that uses instruments and sensors to collect real-time data in video games (34, 35) including data related to the game, and events in which the gamers participated, and the actions they performed throughout the game. This data is commonly used in game design helping designers to understand how gamers interact with the game, which can help them make informed decisions about game usability, playability, and difficulty levels (36). In esports – defined as a sport mediated by an electronic apparatus, where the player intentionally and competitively plays video games, involving spectators, organizations, tournaments, and other infrastructures necessary for the execution and broadcast of a sport (37); telemetry data is especially valuable for analyzing the performance of gamers and teams, as it can provide insights into gamer behavior and help coaches and analysts improve performance and decision-making in real-time during tournaments and matches (1, 38). Beyond these purposes, it can potentially help analyze the gamer from other perspectives and help health professionals detect problematic gamer behaviors (39, 40). However, its potential to help analyze problematic gaming behaviors remains underexplored. A recent study (31) integrated in-game events and gamer behaviors with psychological measures captured with self-reported ratings to examine the relationship between the time spent playing video games and overall well-being.

Another essential component is understanding gamer’s behavior based on their emotional functioning. Recent studies reveal that gamers with lower self-control, characterized by difficulties in regulating emotions, behaviors, and impulses, tend to exhibit a heightened motivation for video gaming, positively correlated with IGD (33, 41). Understanding how the criteria of IGD relate to emotional states is essential for accurate diagnosis and assessment (32). It provides insights into how gaming impacts emotional regulation and deepens our comprehension of the emotional functioning of gamers with problematic behaviors. This could help to understand the transition from regular to problematic behaviors and inform interventions focused on healthier ways to cope with emotions.

Our work introduces a novel method based on players’ gaming data for helping healthy professionals in diagnosing IGD, specifically telemetry data, and emotion regulation data from online gaming platforms, and video data. This approach, which includes precise metrics and visualizations to analyze player gaming behaviors and emotional states, is important to assist health professionals in identifying specific diagnostic criteria of IGD, such as time spent playing and emotional fluctuations. By addressing some of the challenges associated with traditional IGD assessment methods, our research could have an impact on the field.

## 2 Methods

In this section, before detailing the proposed method, we explain how our methodology can be integrated into an existing IGD intervention protocol to illustrate its practical application by health professionals.

### 2.1 Proposed method and intervention protocols of IGD

Our ourk aims to develop tools that could be used in current intervention protocols, such as one of the most widely used and researched interventions for IGD, the cognitive behavioral program PIPATIC (Programa Individualizado Psicoterapéutico para la Adicción a las Tecnologías de la información y la comunicación) (23, 42).

In short, generally at the beginning of the treatment, the therapist conducts an assessment of the patient, consisting of questionnaires (such as IGDS9-SF (25), for IGD diagnosis), interviews, among others, to understand the patient’s life history, symptoms, and diagnosis. The professionals and the patients meet every few days or weeks, where therapists monitor their patients’ progress and symptomatology with questionnaires or follow-up questions. In the case of gaming addiction treatment, monitoring the progress helps the therapists understand the extent of gaming behavior since the last session, the impact of gaming on daily activities, and the patients’ emotional state. Initially, sessions are scheduled more frequently, typically on a weekly basis, and as treatment progresses, sessions may gradually be spaced further apart.

By leveraging the proposed tools, clinicians can conduct thorough assessments, monitor patients’ progress, and deliver more targeted interventions for individuals seeking support for gaming-related concerns. For instance, these technological solutions could enable therapists to analyze the historical gaming behavior of patients (gamers) during initial consultations. By providing access to historical data, therapists can gain insights into the patients’ gaming patterns, including frequency and duration of gaming sessions. This will facilitate the initial diagnostic process and help therapists identify potential signs of gaming disorder.

Moreover, it analyzes emotional fluctuations to provide relevant information for proper diagnosis and therapy monitoring. Hence, we also gathered data on emotion from video faces in which the gamers show their faces during the game and transform them into interpretable features.

Additionally, in each therapy session, it is crucial for therapists to review the patients’ most recent gaming activity. Therefore, the inclusion of indicators, measurements, and visualizations will allow therapists to review the gaming activity and the session intensity from the last consultation (some days or weeks ago), as well as the emotional fluctuations during the week. This monitoring capability will enable therapists to assess changes in gaming behavior over time and could inform treatment planning and adjustment.

Furthermore, the designed visualizations and indicators aim to provide valuable support to the questions of one of the approaches followed in clinic practice, such as those from the self-report questionnaire IGDS9-SF. Analyzing, for example, the evolution of gaming time, we can determine if gaming has become the dominant activity in the daily life of the patients or if they have given up other activities due to gaming, which is one of the nine diagnostic criteria of the DSM-5 (19). Following this, the therapists can identify potential inconsistencies between the responses provided in the questionnaire and the actual reality observed through telemetry and emotion analysis. These disparities may relate to additional diagnostic criteria outlined in the DSM-5, such as deceiving family members or therapists about gaming duration. For example, the patients’ diminished perception of time could elucidate the disparity between questionnaire responses and telemetry data, indicating that the gamer may be unaware of the duration spent gaming.

In conclusion, the proposed solution, explained in the following section, is based on the definition of those metrics and visualizations, their usability, and their direct application to the main IGD diagnosis tool, the IGDS9-SF questionnaire.

### 2.2 Methodology

The proposed approach investigates quantifiable gaming behavior metrics using automated, unobtrusive, and easily accessible methods. Our approach encompasses analyzing behavioral telemetry and facial expressions, aiming to identify gamers’ behavior patterns and examine correlations with IGD. We propose to innovate by exploring telemetry data collected from online game platforms to provide objective in-game gamer behavior, a historical view of gaming behavior, and precise insights into gamer behavior to discover or corroborate patterns of behavior that may otherwise be difficult to recall or identify; and provide data that could be correlated with other measures like, gamer emotions automatically extracted during the game. To accomplish the objective of identification of emotional states, our approach involves examining gamers’ facial expressions during gaming sessions. These measures will also correlate with gamer behaviors and events derived from telemetry data. This will help elucidate how in-game actions are associated with emotional responses, clarifying the relationship between gamer emotion regulation and IGD.

**Figure 1:**
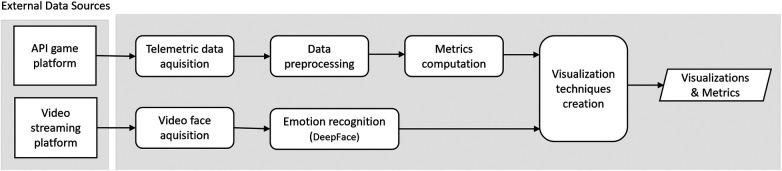
Steps of the proposed method.

The proposed method follows a methodology based on the Knowledge Discovery process through game data and gamers’ video faces (1) and comprises several steps (see Figure 1), namely Telemetric data acquisition, Video face acquisition, Metrics Calculation, and Emotion recognition and the output that are the Visualizations and metrics.

Our approach prioritizes a user-centric design^1^ to address the needs of healthcare professionals. Moreover, our team includes a Clinical Psychologist who works with gaming-related issues in clinical and research settings, ensuring a comprehensive understanding of their needs. She is also the coordinator of the Social Responsibility and Health Department, and of the Research Area at the Portuguese Esports Federation (FPEsports),^2^ and she is also a gamer. She played a crucial role in defining attributes, validating metrics, and analyzing the visualizations during the method’s phases. Leveraging her firsthand gaming experience, she provided valuable insights that enriched the design process and ensured that the tools effectively meet the needs of healthcare professionals.

The main steps shown in Figure 1 involved in managing and utilizing gaming telemetry and video data for analysis and decision-making purposes (1) were the following:

• Telemetric data acquisition and Video face aquisition: Firstly, it is essential to select the information needed to collect and define objective measures (metrics or indicators) that can be extracted from the data to quantify specific aspects of gaming behavior, such as the number of matches per month. It is crucial to analyze the various possible external sources to gather the data within a game environment, including player interactions, in-game events, match dates, or video recordings.
• Data preprocessing is an important step in the data processing pipeline, involving several tasks to ensure that the raw data collected is clean, accurate, and ready for analysis or for the Metrics calculation step. This can involve cleaning and filtering the data to remove errors, missing data, outliers, or irrelevant information (1). Also, this step is responsible for the data storage in databases or data files, locally or in the cloud.
• In the Metrics computation and Emotion recognition phases, we identified relevant measures from the telemetry game data and gamers’ video faces that are indicative of gamer behavior and developed the respective calculation methods using statistical analyses and related techniques. The selection of these measures was based on their relevance and predictive power and may include variables such as total gaming duration, frequency of specific in-game actions, number of matches per day/week/month/weeks/year, and emotional states experienced during gaming sessions. To identify the emotional states experienced during gaming sessions (e.g., anger, fear, neutrality, sadness, disgust, happiness, and surprise) we analyzed gamers’ video facial expressions using the DeepFace software.
• Visualization techniques creation. To enable the extraction of insights and patterns from the processed data and to gain a deeper understanding of gamer behavior and correlate some game events with emotional responses during gameplay, it is important to create visualization techniques, such as charts, graphs, and tables. These visualizations represent the findings in a meaningful and accessible way in order to help health professionals interpret the results and derive actionable insights.

Concerning the analysis of the various possible external sources to gather the data within a game environment, our method can be applied to any video game chosen. Nevertheless, the game genre and the game must be chosen to ensure the relevance and effectiveness of the data collected for analyzing gaming behaviors, particularly those related to IGD, and it is important to follow these guidelines:

1. Choose the game genre based on their relevance to IGD: a recent study (16) reported that playing massively multiplayer online role-playing games, first-person shooters, and real-time strategy games/Multiplayer Online Battle Arena is associated with more time spent gaming and higher endorsement of IGD symptoms.
2. Choose popular and widely played games. Popular games are more likely to attract gamers and increase engagement during gaming sessions.
3. Choose games with supportive and accessible communities open to research collaboration.
4. Prioritize games for which the gaming platforms provide services to access telemetry data with automated and publicly accessible methods.

In the following subsections, we provide a detailed account of the data acquisition and preprocessing stages, definition and calculation of the metrics, and illustrate the application of our method using a specific game and data from four gamers. These use cases showcase the metrics and visualizations, and demonstrate how our method is applied.

#### 2.2.1 Telemetric data acquisition and video face aquisition

This step of the proposed process corresponds to selecting the information needed to collect and define objective measures (metrics or indicators). The data we want to gather is related to telemetry data, such as the list of matches played by the gamer. We want to extract information that can be useful to determine how many matches s/he played in the last days and their duration. Additionally, we process video information to extract gamers’ emotional states, such as anger, happiness, or surprise. This section starts with an explanation of the chosen game, followed by an explanation of how we extracted data from the different environments: gaming and video data.

We chose Counter-Strike^3^ (CS) from Valve following the exposed guidelines, namely it is a game of the family of first-person shooters, and CS is a game for which automated and open services exist to access telemetry data, and as a popular and widely played game, it was easier to find public face recordings. Shortly in CS matches, each game consists of multiple rounds where teams (with five gamers each) compete against each other. The number of rounds varies depending on the game mode being played, and in the classic mode, which is the most common, a match typically comprises 30 rounds. Each round typically lasts around 1 min and 45 s to 2 min, contributing to a total match duration of approximately 30 to 45 min. The team that wins 16 rounds first is declared the overall winner of the match.

In order to demonstrate the application of our method and better understand the proposed metrics and visualizations, we extracted information from four gamers as use cases. The four gamers, two amateur (G1 and G2) and two professional, G3 and G4 (e.g., earn money from donations during their broadcasts and devote most of their time to gaming), were chosen to provide a diverse perspective on gaming behaviors across different skill levels.

It is worth noting that we only used publicly available gaming data from both platforms, ensuring the confidentiality of players’ information and the protection of their privacy in accordance with best practices. This approach respects the principles of fair use and promotes the public interest.

**Telemetric data acquisition.** We initially considered Steam^4^ as a primary source for telemetry data due to its popularity among gamers, particularly for playing CS. However, it lacked player-specific information and statistics. One valuable data source explored was the Steam login history,^5^ which could provide insights about gaming time, but access to this data requires gamer permission, and one fundamental requirement of our work is the open accessibility to the telemetry game data. We decided to use the FACEIT^6^ platform to extract the telemetry data for the four gamers. Like many online gaming platforms, FACEIT typically provides information such as a gamer’s username, gaming statistics (e.g., win/loss ratio, kill/death ratio), match history, and achievements. However, we do not have access to personal information except some basic profile information that the player chooses to share publicly. This platform provides all the necessary information through their public API^7^ and includes information about gamers, matches, and tournaments. For each gamer, we collected information about all the matches between January 2021 and December 2022, namely the start time and duration of each gaming session (*starting and ending datatimes*) allowing us to calculate the duration of matches, the *day part* and *weekday* of every match, the continued *gaming time*, and other player metrics. In addition, we also gathered in-game actions performed by the player aggregated in detailed match statistics, for instance, the number of *kills*, *deaths*, *assists*, or *kill-death ratio*, among others.

The rationale behind acquiring two years of historical data stems from the crucial role of seasonality. This duration enables us to thoroughly examine and explain individual behaviors during particular periods throughout the year. For instance, we can discern patterns such as fluctuations in gaming activity during holidays or exam periods for a student gamer, thereby providing valuable insights and justifications.

**Video face aquisition.** Furthermore, Twitch^8^ was utilized to obtain live-streaming video content, particularly from the two professional gamers, for subsequent analysis and emotion extraction. Twitch is a widely-used online platform, attracting a diverse audience of active gamers who stream digital video broadcasts and passive viewers. Each video was selected based on specific criteria, ensuring clear frontal visibility of the face, proper lighting conditions, and close camera proximity. This selection process aimed to streamline the subsequent video analysis phase. For the two amateur gamers, it was not possible to obtain this data.

To identify the emotional states experienced during gaming sessions (e.g., anger, fear, neutrality, sadness, disgust, happiness, and surprise) we analyzed gamers’ video facial expressions using the DeepFace software library.^9^ DeepFace, developed by Facebook, is a system designed for facial analysis and emotion recognition tasks. It leverages deep learning techniques to accurately detect faces, estimate facial attributes, and recognize emotional states from images or video frames. For the two professional gamers, we collected several videos containing several consecutive matches (5 matches on average and with a duration of 4–5 h) during 2 weeks. This data range is different from telemetry data because it is not possible to know the actual date of the videos loaded more than a month ago. The Twitch platform only informs how many months ago the video was uploaded.

#### 2.2.2 Data preprocessing

The data we collected from the mentioned platforms did not require a cleaning process due to the good state of the main data sources from FACEIT and Twitch.

#### 2.2.3 Metrics computation and emotion recognition

In this section, we present the metrics computation phase of the proposed method derived from the raw data collected from the FACEIT and DeepFace video analyses.

Telemetry gaming data: As noted earlier, our data extraction process relied on FACEIT to obtain information about gamers and their gaming activities. We extracted over 50 variables (features) from this platform, with key variables including *started_at* and *finished_at*, and the *match_id*. Additionally, we extract some important gaming activities, namely *health_points* and if the gamer *is_alive*, from player-frame granularity; and *tick* and *attacker* from kill granularity.

These raw data variables were aggregated and combined to create more intricate features that provided additional value. Below, we explain the definition of the most important:

• A session represents a series of consecutive matches played by a gamer, starting when they begin playing and ending when they stop. Consecutive matches are those played within 30 min of each other, determined based on the team gaming experience and the distribution of the *time between matches* variable (60th percentile). Each match belongs to only one session, but multiple matches may be part of one session. Multiple sessions can occur within a day.
• Gaming time refers to the total duration of a gaming session, which includes the time between the first and last match.
• Based on the match date and time, we defined two new features: day part and weekday. *Day part* can take three values: morning (from 6 a.m. to 1 p.m.), afternoon (from 2 p.m. to 9 p.m.), and evening (from 10 p.m. to 5 a.m.). *Weekday* indicates if the match is played during the midweek (from Monday to Friday) or during the weekend (Saturday and Sunday).

From these features, we define the metrics presented in Tables 2–4. The most relevant is the amount of time spent gaming each month (*Avg played hours per month*), the number of matches played per session (*Avg matches per session*), and the average duration of each gaming session (*Avg hours per session*).

Video emotion data: We examined the emotions per second in the numerous videos of G3 and G4, capturing the frequency of each emotion experienced, including anger, happiness, fear, sadness, disgust, surprise, and neutral. To improve clarity and facilitate comprehension of the gamers’ emotions, we opted to smooth the trends of each emotion by calculating the median of the previous five seconds for each instance. This approach facilitated the identification of emotions such as happiness or anger, making them more discernible.

**Figure 2:**
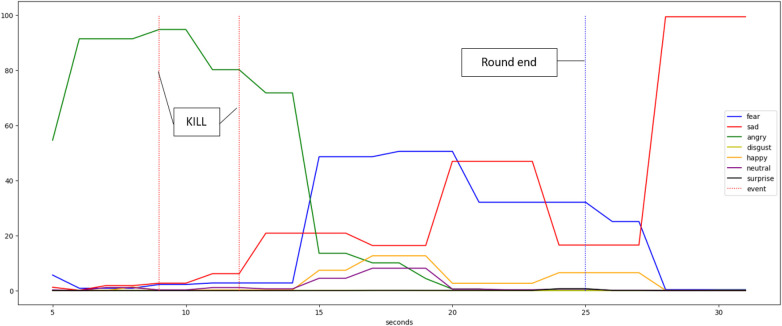
The percentage of time experienced by Gamer 4 for each emotion per second during one round, highlighting three key events: two kills and the end of a round.

Furthermore, we aim to understand the dynamic changes in emotions throughout a gaming session. To achieve this, we correlate in-game telemetry data with emotions extracted from video analysis. The objective is to examine the player’s emotional state during pivotal moments such as kills, deaths, and the initiation or conclusion of rounds. An illustrative example of this analysis is depicted in Figure 2, where it is evident that G4 experienced anger upon eliminating two opponents but exhibited fear and sadness at the round’s conclusion. This correlation underscores the need for further investigation in future studies. While this approach, involving the analysis of emotions by player, event, round, and match, is intricate and challenging to execute, one viable solution is to analyze the median emotional response of players across events and matches, as demonstrated in Table 1. This table displays the median emotions experienced during instances such as kills, deaths, and the start and end of rounds within a single match. In kills and deaths events, emotions are calculated by taking the median values (in percentage) of emotions 3 seconds before and after the event.

**Table 1:** Median emotion level felt during each event.

Event	Angry	Disgust	Fear	Happy	Sad	Surprise	Neutral
Kills	13.54	0.05	11.23	2.14	8.92	0	0.63
Deaths	22.56	1.28	17.47	2.39	27.86	0.04	2.78
Round ini	37.83	0.02	36.58	0.04	17.75	0.02	2.4
Round end	0.07	0	32.04	2.65	46.9	0.06	0.3

**Figure 3:**
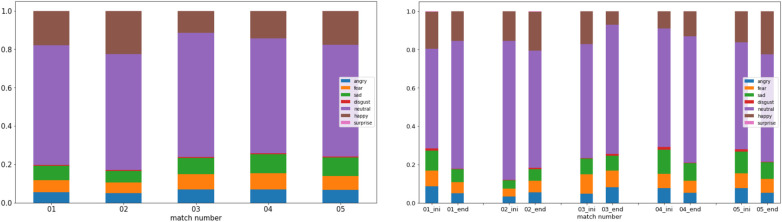
Percentual emotion average felt (left) during 5 matches separated by sessions and (right) on the first and last 5 min during 5 matches of G3.

After conducting several tests, we determined that calculating the average emotion experienced throughout the match yielded the most effective results. This approach involved computing the mean of each emotion across the entire duration of the match, disregarding specific events. As shown in Figure 3-left, our analysis showcases the emotional experiences of G3 across five distinct matches within a single session. It provides a comprehensive view of the overall emotions experienced by gamers throughout each match, disregarding any sudden emotional changes that may occur during gameplay. On the other hand, it is crucial to understand whether external factors or events, unrelated to the game, influence the players’ emotions, such as household or professional environments. Therefore, we have created a chart that shows the gamer’s emotional state at the start (first 5 min) and at the end (last 5 min) of the match, as illustrated in Figure 3-right. We detail this type of visualization in the next section.

#### 2.2.4 Visualization techniques creation

This phase of our method involves creating appropriate visualization techniques to represent the processed data in a meaningful and accessible manner. We developed various charts, graphs, and tables, which will be presented in the subsequent section. As previously mentioned, the visualizations were selected in collaboration with a healthcare professional to ensure that the tools effectively meet their needs.

## 3 Results

In this section, we analyze how each developed metric and visualization can assist therapists in the diagnosis of IGD. It is organized into three subsections. The first focuses on the initial stage of the treatment, where the therapist starts knowing the patient, as explained in Section 2.1. To achieve this, the therapist needs to understand the historical behavior of the gamer. The second section, recent gaming behavior, involves monitoring the therapy and analyzing its effectiveness. Prior to each consultation, it is helpful to briefly review the patient’s behavior in the past few days or since the last visit, including analyzing gaming telemetry and emotions data. The last section, recent emotional behavior explains the metrics and graphs regarding emotional fluctuations that the therapists could explore just before each consultation.

### 3.1 Historical gaming behavior

The literature suggests that therapists must have a clear understanding of their patients’ situations from the outset. This entails examining the duration of gaming sessions on a daily, weekly, and monthly basis, as well as identifying the specific times of day during which individuals typically engage in gaming activities. Additionally, is equally essential to assess potential disparities between weekend and weekday gaming behaviors. To accomplish this goal, we propose a set of indicators that can help understand the historical gaming behavior (Table 2), such as the *number of matches* or the *total hours played*. Also, the intensity can be analyzed through the *average hours per session* (all the games played with less than 30 min of difference between them) or compare the gaming behavior by weekday and part of the day.

**Table 2:** Main telemetry metrics for the four gamers.

	G1		G2		G3		G4	
	2021	2022	2021	2022	2021	2022	2021	2022
**General stats**								
Number of played matches	320	402	570	499	488	1,178	805	1,009
Average match duration (min)	40	40	40	40	40	40	40	40
Total hours played	223	281	390	344	328	816	570	731
Total hours connected to FACEIT	252	316	438	389	375	967	699	892
Number of sessions played	170	227	314	273	253	410	151	193
**Session stats**								
Avg number of sessions per week	5.21	5.22	6.8	6.17	5.8	8.67	3.48	4.59
Avg number of sessions per month	17.1	20.82	26.58	23	21.25	34.25	12.58	17.82
Avg matches per session	1.86	1.77	1.82	1.83	1.93	2.87	5.33	5.23
Avg hours per session	1.47	1.39	1.4	1.42	1.48	2.36	4.63	4.62
**Monthly stats**								
Avg matches	32	36.55	47.5	41.58	40.67	98.17	67.08	91.73
Avg played hours	22.34	25.55	32.53	28.67	27.32	67.97	47.46	66.44
Avg matches during midweek	23	27.27	35.08	31.42	33	76.08	52	72.45
Avg matches during weekend	11.25	9.27	13.55	11.09	10.42	24.09	16.45	19.27
Avg hours played during midweek	16.14	19.17	24.28	21.56	22.31	53.12	36.89	52.65
Avg hours played during weekend	7.75	6.37	9.01	7.75	6.87	16.2	11.53	13.79
**Weekly stats**								
Avg matches per week	8.65	7.58	10.56	9.24	9.96	21.42	16.77	20.18
Avg played hours per week	6.04	5.3	7.23	6.37	6.69	14.83	11.86	14.62
Number of weeks played	34	46	49	47	44	49	44	44
**Day part stats**								
Avg daily morning hours	0	0	0	0	0.04	0.02	0.09	0.03
Avg daily afternoon hours	0.06	0.08	0.15	0.08	0.51	0.85	0.56	0.87
Avg daily evening hours	0.55	0.69	0.95	0.87	0.38	1.39	0.89	1.04
Morning matches percentage	0	0	0	0	0.05	0.01	0.06	0.02
Afternoon matches percentage	0.1	0.1	0.13	0.09	0.55	0.38	0.37	0.45
Evening matches percentage	0.9	0.9	0.87	0.91	0.41	0.62	0.58	0.54
**Weekday stats**								
Weekend matches percentage	0.28	0.25	0.26	0.24	0.26	0.22	0.22	0.21
Midweek matches percentage	0.72	0.75	0.74	0.76	0.74	0.78	0.78	0.79

The analysis of these indicators for the four gamers reveals that in 2021 and 2022, G1 and G2 (both amateurs) primarily played in the evening, possibly in their free time after school/work. These gamers seem to maintain their gaming pattern during the period of 24 months, without significant changes. The professional gamers, G3 and G4, had a more balanced day-part ratio, which was expected since its their job. Overall, the four gamers played approximately 75% of their games during weekdays (midweek hours accounted for 72% of the total week time) without significant changes between 2021 and 2022. Regarding the *average hours per session*, G4 seems to be an intensive player, as they appear to maintain a pattern of long gaming sessions (approximately an average of 4 h per session) during 2021 and 2022. The other professional player, G3, has doubled its playtime in 2022.

**Figure 4:**
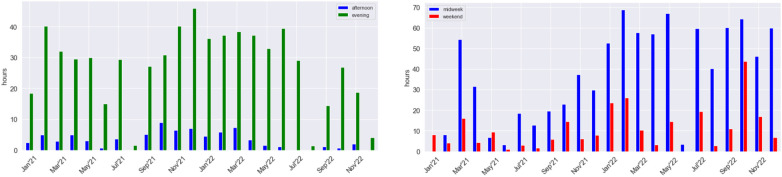
Total monthly hours played in the afternoon and evening by Gamer 2 (left) and during midweek and weekend by Gamer 3 (right) during two years.

To facilitate the analysis of time spent playing in each part of the day, we developed the visualization shown in Figure 4-left. The bars corresponding to the morning hours played were minimal for all gamers; therefore, they were not represented in the graph. The data shown covers 24 months from January 2021 to December 2022. This graph corroborates Table 2, where G2 plays mainly in the evening. Typically, they spend 30 or more hours playing during the evening every month. However, they do not play in August, possibly due to vacation time. The evolution of the *average hours played per month* is relatively stable, except for the last months when they reduced the total playtime.

**Figure 5:**
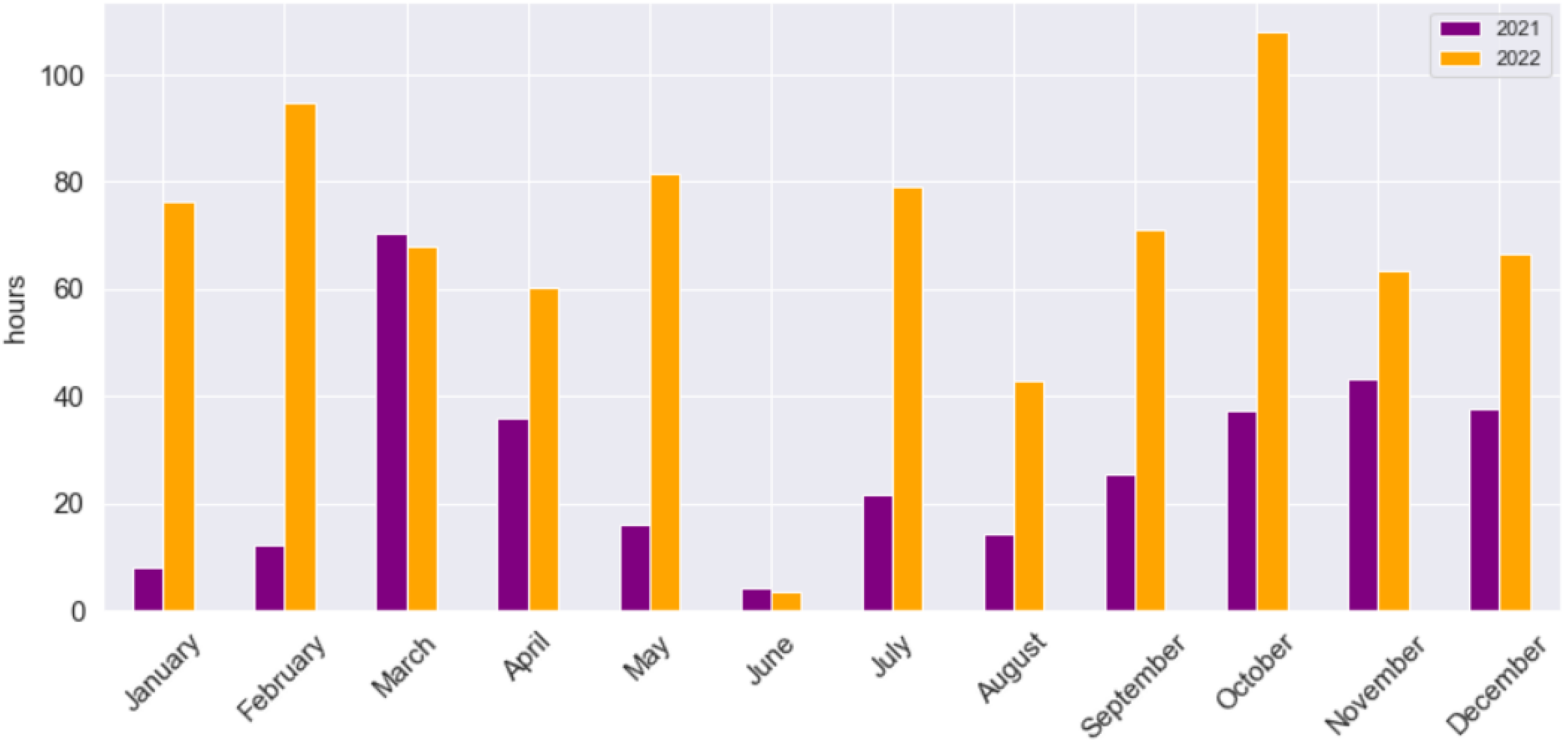
Total monthly hours per year for the Gamer 3.

To explore variances in gaming duration between midweek and weekend sessions, we developed the visualization depicted in Figure 4-right. Once more, we can track the gaming behavior of G3. Throughout 2021, except for March, the average monthly playtime was around 27 h (see Table 2). However, in 2022, the playtime more than doubled, reaching 67 h. This substantial increase may indicate a shift in gaming behavior or a potential transition to professional gaming. Notably, there is one month where the player reduced gaming time, prompting further analysis into the underlying reasons. To investigate potential seasonal patterns, we constructed the chart depicted in Figure 5. This chart illustrates the total monthly gaming hours by year. Interestingly, a recurring pattern emerges where G3 pauses gaming activities every June. Furthermore, most yellow bars surpass their purple counterparts, indicating a substantial increase in gaming time during 2022.

**Figure 6:**
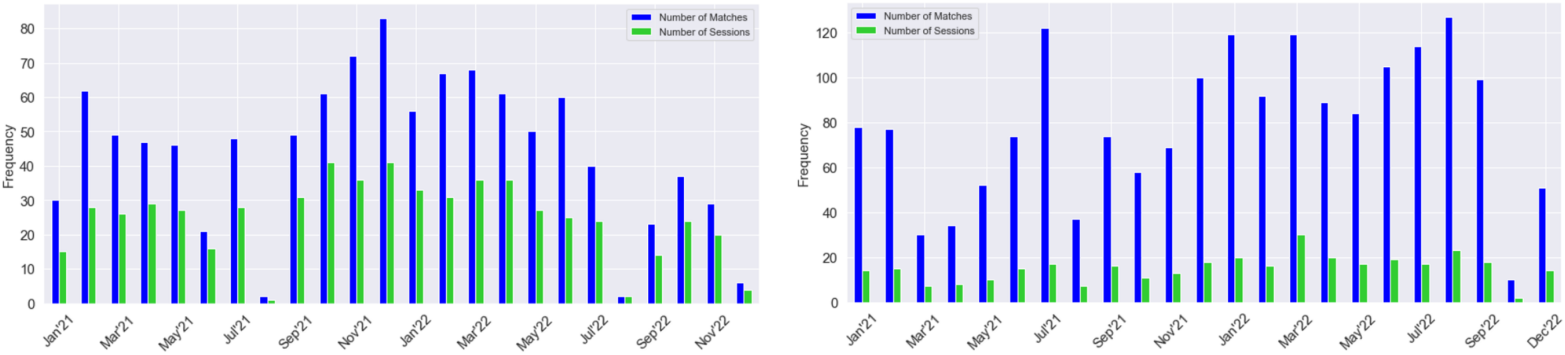
Monthly number of matches vs. number of sessions for Gamers 2 (left) and 4 (right).

To measure the gamer’s intensity and an accurate overview of their gaming habits, we analyzed the duration of gaming sessions and developed the graphs shown in Figure 6. The blue bars indicate the number of monthly matches, while the green bars represent the number of gaming sessions played per month. If a gamer only plays one match per session, the blue and green bars will be of equal height. However, the bars will be significantly far apart if the gamer plays all the monthly matches in just a few sessions. Therefore, the greater the difference between the number of matches and sessions, the more intense the gamer’s gaming behavior. For example, if we analyze the number of matches and sessions, we can infer that G4 is a very engaged gamer.

The information from the various visualizations (graphs and tables) can help the health professionals to conduct further analyses of the gamers. For example, G4 played 67 matches per month on average in 2021 and 91 in 2022, increasing by 35%. Nevertheless, the number of matches per session remained stable, around five, and the intensity stayed equal (see Figure 6-right). In contrast, G2’s playing time remained stable from 2021 to 2022 (around 1.8 h), and their sessions on average took 1.4 h (1 h 24 min), less than half (see Table 2, Figure 6-left).

### 3.2 Recent gaming behavior

Monitoring patients weekly to follow their initial diagnosis is crucial. To assist therapists in this task, we created specific indicators for tracking their gaming habits between consultations. These indicators are intended to be presented to therapists before each session, offering insights into the patients’ recent gaming behavior. The metrics available for therapists are presented in Table 3, as well as the values for the four gamers. The metrics include the *total number of hours spent in the past 7 days* and the *percentage difference compared to the previous week and month*. From the values we could infer that G1’s gaming time has seen a notable increase of 52% over the past 7 days but decreased by 11% compared to the previous month, indicating a less stable trend in gaming behavior. For G2, we can identify an increase of 72% in gaming time over the past 7 days, while G3 decreased by 15% in playing time compared to the previous week and by 39% compared to the previous month. These changes could be used by the health professionals to further understand the recent behavior of the patients.

**Table 3:** Metrics related to the gaming time during the last 7 days, compared with the previous week and month.

Time played	G1	G2	G3	G4
Total hours last 7 days	8.2	11.2	14	21.4
% Difference last week	52	72	−15	5
% Difference last month	−11	6	−39	−9

Furthermore, the therapists can also evaluate the frequency and duration of the gamers’ sessions in the previous week (*total sessions last 7 days*) and the percentage difference compared to the previous week and month with the metrics presented in Table 4. From this information, an health professional could infer that G1 has been exhibiting an inconsistent gaming behavior lately. They played 7 times during the week, with a ratio of 1.2 hours per session, resulting in an increase of 9% compared to the previous week but a decrease of 14% compared to the previous month. On the other hand, for G2, G3, and G4, the therapists could infer that patients played less than their usual, showed by a decrease of hours/session compared to the previous week and previous month.

**Table 4:** Metrics related to the intensity and duration of the sessions during the last 7 days, compared with the previous week and month.

Intensity	G1	G2	G3	G4
Total sessions last 7 days	7	10	9	9
Hours/session ratio last 7 days	1.2	1.1	1.6	2.4
% Difference hours/session last week	9	−15	−43	−29
% Difference hours/session last month	−14	−27	−16	−43

**Figure 7:**
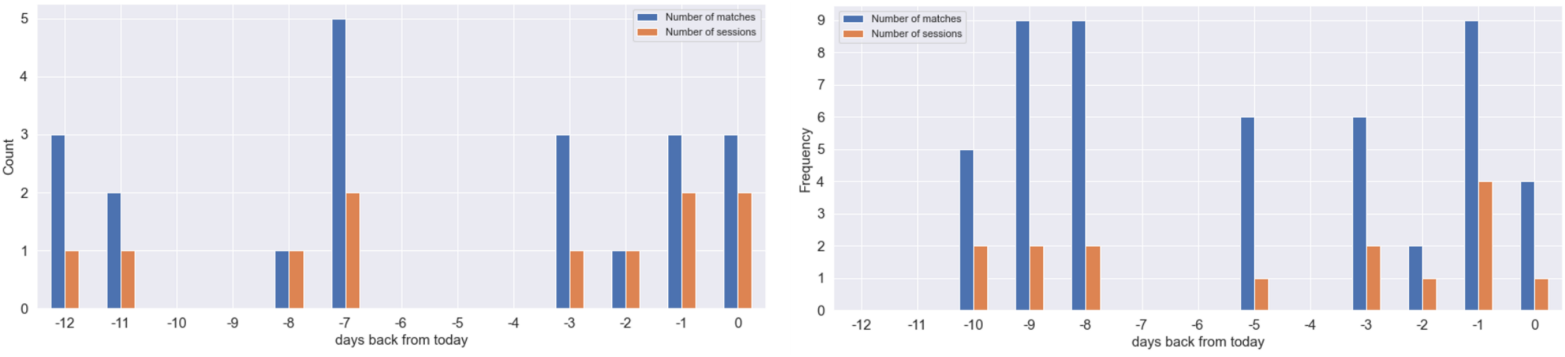
Daily number of matches vs the number of sessions during the last 2 weeks for Gamer 1 (left) and Gamer 4 (right).

To complement the metrics information, we developed the plots presented in Figure 7 where the vertical axis displays the number of matches (represented by blue bars) and sessions played (represented by orange bars) per day, spanning from the current day to 12 days prior. As we saw in Figure 6, also in this figure, a wider disparity between the blue and orange bars suggests more intense gaming sessions. We can see that over the past 13 days, G4 engaged in 9 matches on three occasions, implying gaming sessions of over 6 hours per day (across 2 or 3 sessions), representing the days where they played the most. In contrast, G4 did not played for five days in the past 13 days, and only engaged in a session with two matches in one of the other days. On the other hand, G1 played only 3 matches on most days, equivalent to 2 hours per day, sometimes in more than one session. The gaming behavior of both gamers appears consistent across the two observed weeks.

### 3.3 Recent emotional behavior

**Figure 8:**
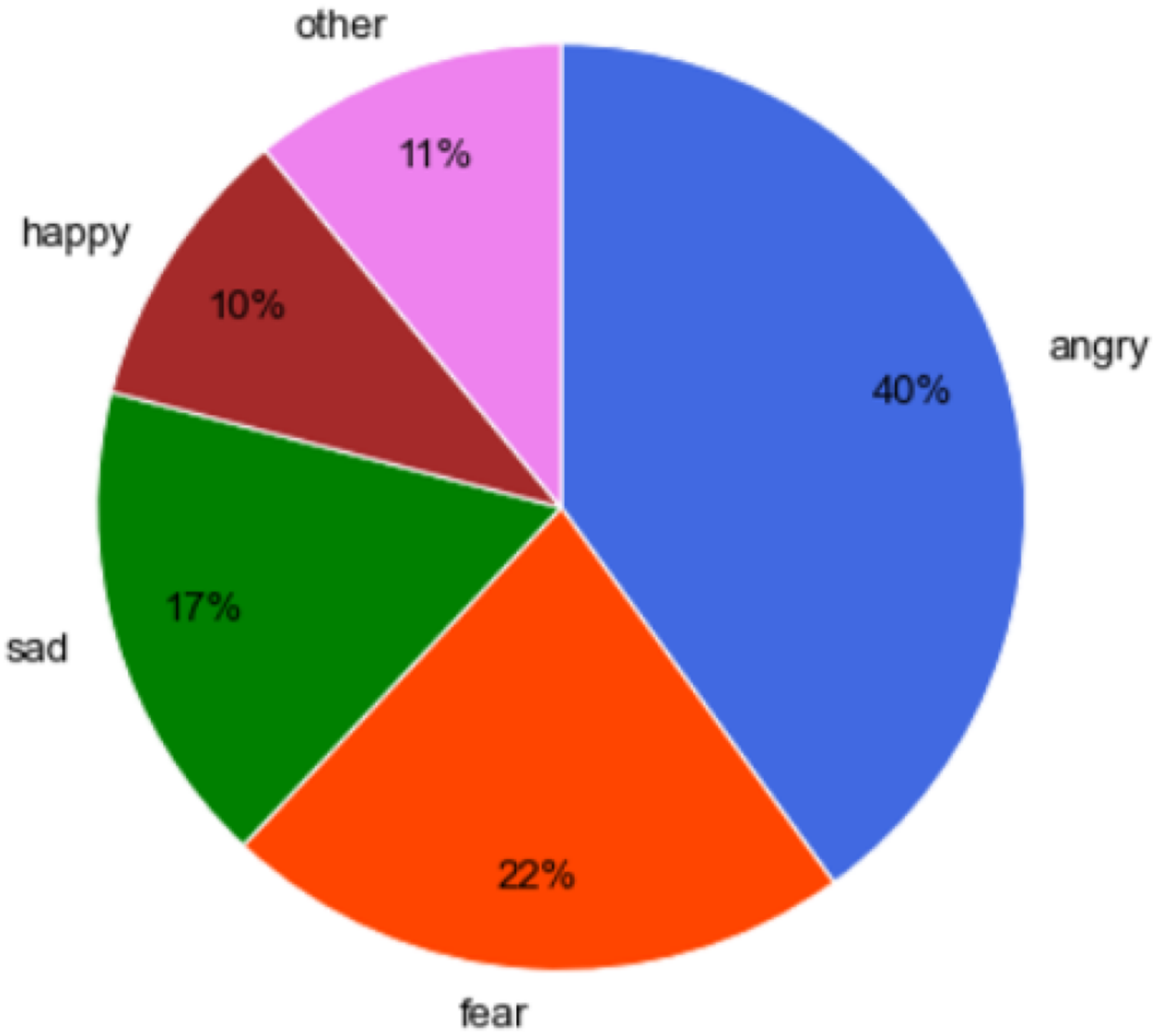
Percentual average emotion felt by Gamer 4 during the last 2 weeks.

To allow therapists to explore gamers’ emotional fluctuations, we developed a pie plot (Figure 8) with the percentual average emotion felt by a gamer during the last 2 weeks. From the example in Figure 8, the therapists can see that G4 felt mostly negative emotions, namely angry, fearful, and sad, 41%, 21%, and 18%, respectively.

**Figure 9:**
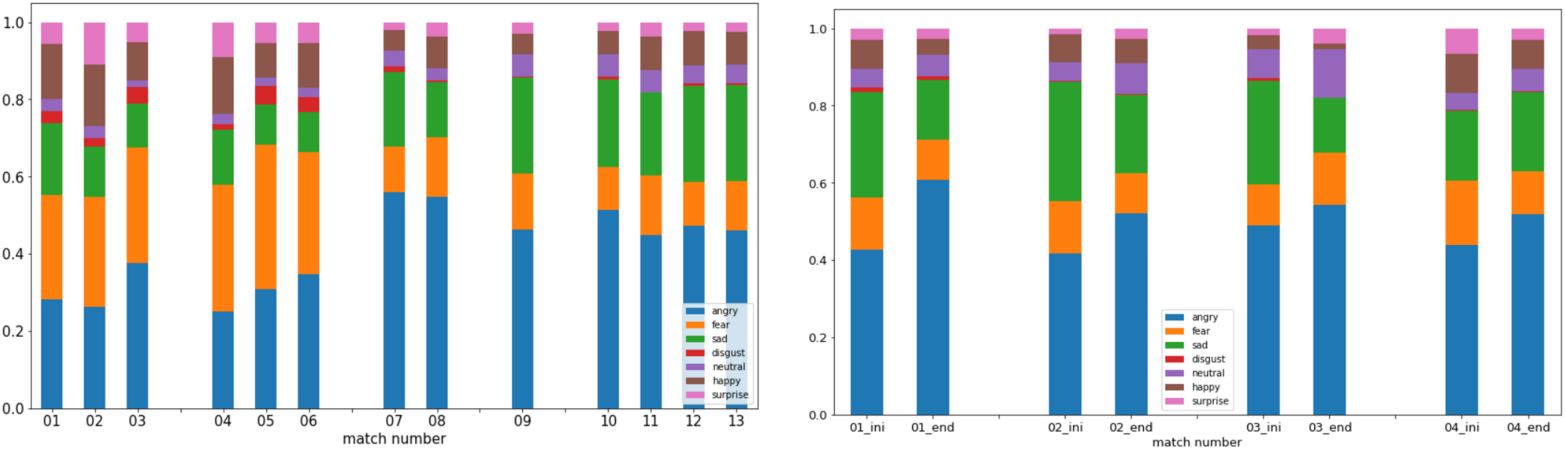
Percentual emotion average felt during (left) 5 matches of Gamer 4 and separated by sessions; and (right) on the first and last 5 min of each match during the last session (4 matches) of Gamer 4.

To understand the variation between matches and determine whether the emotions experienced are constantly present or it is only noticeable at certain moments, we developed the visualization in Figure 9-left, where the focus is on match-by-match emotional behavior, with the space between them being used to separate different sessions. Again, negative emotions, such as anger, fear, and sadness, appear to be predominant during every match of G4. However, there are some differences between sessions. Fear and anger are the most significant emotions in the first two (6 matches). Besides, in the rest of the sessions, anger became even more dominant, and sadness overtook fear in importance.

Another important analysis is considering whether emotions change throughout a gaming match or are influenced by external factors. We designed the graph shown in Figure 9-right that can help to answer these questions. Just like in Figure 3-right, we can observe the emotions experienced during each match’s first and last five minutes. Figure 9-right represents the last four matches analyzed in Figure 9-left. We can observe how the state of mind of gamers can change. For example, for gamer G4, as previously mentioned, anger is the most common emotion observed. Furthermore, it appears to increase as the match progresses. Anger levels are lower in the first five minutes than in the last five minutes. Conversely, sadness decreases toward the end of the match. Although happiness levels are low, they also seem to decrease at the end of some matches. Meanwhile, G3 shows a calm mood while playing, even happiness (see Figure 3-left).

## 4 Discussion

In this section, we discuss how clinicians can use the proposed method’s results (metrics and visualizations) in conjunction with the nine answers to the IGDS9-SF questionnaire for patients that they suspect of problematic gaming behaviors. They already know what to look for and what relevant behavioral indicators to monitor. In particular, our approach will help clinicians to identify disparities between the metrics and visualizations and the responses provided by gamers to the questions within the IGDS9-SF questionnaire. Given the nature of the questions, it is important to note that while telemetry data and emotional states can provide important insights into gaming behavior, they cannot directly address the subjective experiences described in questions such as 1, 6, and 7. These questions delve into the individual’s feelings, perceptions, and behaviors, which cannot be captured by those metrics and visualizations.

For example, in Question 1 (“Do you feel preoccupied with your gaming behavior?”), telemetry data might reveal extensive gaming activity, but it cannot directly measure the individual’s sense of preoccupation with gaming. However, by examining trends in gaming behavior over time, therapists can better understand the extent to which gaming occupies the individual’s daily life and assess whether this preoccupation aligns with the subjective experiences described in this question. For instance, observing whether gaming is becoming a primary daily activity of the gamer through the significant increase of the metric *last 7 days total hours* in Table 3.

Similarly, for Question 6 (“Have you continued your gaming activity despite knowing it was causing problems between you and other people?”) and Question 7 (“Have you deceived any of your family members, therapist, or others because of the amount of your gaming activity?”), telemetry data and emotional states cannot provide insight into the individual’s motivations or intentions behind their actions. Therefore, while telemetry data and emotional states can offer valuable information in assessing gaming behavior, they should be complemented with self-reported responses to understand the subjective experiences described in these questions. Next, we discuss the remaining six questions that can be supported by the metrics and visualizations.

**Question 2: Do you feel more irritability, anxiety, or even sadness when you try to either reduce or stop your gaming activity?**

We cannot directly answer this question using the proposed metrics or visualizations. However, we can suppose that if the gamer finishes a game session but shortly afterward starts playing again, it could mean that stopping was difficult, possibly accompanied by negative emotions, such as anger or even sadness.

The therapist can analyze the emotions at the end of each match or session and compare the emotional status evolution over several consecutive matches with the visualization in Figure 9-left. Also, s/he can compare the emotions at the first and last 5 min of each match and observe if the negative emotions have increased during the match (Figure 9-right). If negative emotions are greater at the end of the match compared to the beginning, it could possibly be an indicator of irritability or sadness. The therapist can also analyze the metrics *number of daily gaming sessions*, and recent gaming behavior indicators, such as *weekly playtime* (Table 3) and *weekly intensity* (Table 4) as they can be possible indicators of the difficulty in stopping.

**Question 3: Do you feel the need to spend an increasing amount of time engaged in gaming in order to achieve satisfaction or pleasure?**

The purpose is to assess whether the patient has developed a tolerance to gaming, which may result in increased time spent playing in order to experience satisfaction or excitement.

The therapist can analyze the time spent playing games, mainly if the patient is playing more frequently and for longer sessions, examining recent gaming behavior indicators, and if the time spent on in-game activities has increased or changed in the last few days. To achieve this, s/he can observe Table 3 to analyze if the *total gaming time* has increased regarding the previous week or month; and Table 4 to examine if the *duration of the sessions* is stable or has increased face to last week and month. Analogously, Figure 7 informs about the total number of hours and the intensity of the sessions. If these indicators reveal an increasing tendency regarding gaming time and duration of the sessions, it can mean that the gamer needs to spend more time playing video games and could be developing tolerance to gaming.

**Question 4: Do you systematically fail when trying to control or cease your gaming activity?**

One way to analyze the difficulty of stopping gaming is to observe when the gamer engages in several gaming sessions daily and analyze the total number of hours dedicated to gaming during the last days.

To achieve this objective, the therapists can examine the metrics *total hours in the last 7 days* and the *ratio of hours/session* in the last week (Tables 3, 4). If gaming time and intensity increased during the last week or month, it could indicate that the gamer is not achieving the goal of ceasing gaming activity. Additionally, the therapist can evaluate the relationship between the number of daily matches and the number of daily sessions in Figure 7. If the gamer usually plays more than one session, plays every day, or whose sessions are longer, it could be an indicator of problems with stopping gaming activity.

The therapist can also obtain a global view by analyzing the total time spent per month, by midweek/weekend and by afternoon/evening in Figure 4. If each month’s total gaming time is not being reduced, it could also indicate that the gamer is failing to cease gaming.

**Question 5: Have you lost interest in previous hobbies and other entertainment activities as a result of your engagement with the game?**

A possible way to examine the loss of interest in hobbies and other activities is by observing the amount of time spent over several months from different perspectives, on weekends or part of the day. If a gamer plays a lot during the day and at weekends, it could mean that s/he does not have time for hobbies.

To achieve this analysis, the therapist can examine if there exists a growth in the gaming time by day part (afternoon and evening) and by the weekday (midweek or weekend) presented in Figure 4. However, the therapist needs to know the patient’s working or studying habits in order to interpret these indicators and visualizations correctly.

**Question 8: Do you play in order to temporarily escape or relieve a negative mood (e.g., helplessness, guilt, anxiety)?**

To accomplish the analysis of whether a patient uses gaming to escape from negative situations in their daily life, the therapist could observe the emotional status of the gamer before the session starts and analyze how negative (or positive) s/he feels. Then, compare with the end of the session, concluding the evolution of its feelings, through Figure 9-left. Hence, if positive emotions predominate at the beginning of the session, a possible hypothesis is to reject the relieving negative mood. Otherwise, there is a possibility that the motivation for gaming is to escape from negative thoughts. Furthermore, if the emotions get even worse over several consecutive matches, it can mean that gaming does not help to cope or escape the initial pessimistic scenario. Additionally, the therapist can analyze the emotional status at each match’s beginning and end in Figure 9-right.

**Question 9: Have you jeopardized or lost an important relationship, job or educational or career opportunity because of your gaming activity?**

Similar to Q5, this question aims to determine if gaming impacts other activities, in this case, regarding educational and professional occupations rather than hobbies. We can argue that if gaming activity has increased or the intensity of the sessions is higher, it could be difficult for the patient to engage in any other activity, such as a job or educational courses. To achieve this, the therapist can understand the tendency to spend time gaming, particularly on weekends or midweeks, using the visualization of Figure 4-right. If the gamer significantly increased their gaming time during the last months, s/he probably would have jeopardized his educational or professional career. Another interesting perspective to analyze is the gaming time by part of the day. If the gamer usually plays during work time, it is also possible that s/he negatively affects his professional career. This situation can be analyzed through the Figure 4-left. Once again, to correctly analyze this information, it is important to know the personal schedules of the patient and when s/he usually spends time on those jobs or educational careers.

## 5 Conclusion

This paper introduced a new methodology for analyzing gaming behavior indicators, aiming to enhance the diagnosis and comprehension of IGD. Leveraging behavioral telemetry data and extracting emotional states, the proposed metrics and visualizations provide a nuanced understanding of gamer behavior and emotion regulation. We used data from four gamers as use cases to explain the metrics and visualizations and to demonstrate the application of our method. Our findings outline the potential insights that can be gained from a tool that gathers these telemetry data, emotion regulation data, and gaming patterns. A tool composed of these precise indicators can aid healthcare professionals in diagnosing IGD and monitoring the therapeutic process, potentially helping to resolve some of the problems and difficulties associated with traditional methods of IGD assessment. Additionally, the metrics and visualizations can also inform therapists about each gamer’s problematic behavior and gaming habits, allowing for personalized treatment tailored to the individual and their needs.

It is important to acknowledge several limitations of the proposed approach. First, our results are based on telemetry data from Counter-Strike, which may not fully capture the broader gaming habits of patients with IGD, who often engage with a variety of game genres. Additionally, as this is exploratory research, it lacks comprehensive clinical validation. Lastly, the absence of facial recordings during gameplay limits our ability to analyze emotional data.

Future research endeavors will focus on extending the application of our methodology to facilitate more personalized interventions for individuals affected by problematic gaming behaviors and validation. Expanding data collection to encompass telemetry from various genres and types of video games is essential. Additionally, it is important to develop an interactive and user-friendly application that allows health professionals to designate a specific gamer and subsequently access and analyze the metrics and visualizations presented, allowing a comprehensive understanding of gaming behaviors. This will allow us to validate our method with healthcare professionals to assess the tool’s usability and helpfulness in assisting professionals with tasks such as diagnosis and monitoring of IGD.

Additionally, to mitigate the problem of the absence of datasets involving video gamers, we plan to conduct a longitudinal experimental study with repeated game sessions over several months. This study will include gamers of different profiles (professionals and amateurs, healthy and those with problematic behaviors) to collect video facial data, physiological data, and other relevant information. These datasets will enable us to explore correlations between emotional states, gaming behaviors, self-reported data, and IGD criteria. The overarching aim is to provide clinicians with objective indicators for a more informed assessment of gamers. We also envision extending our method to explore the impact of player interactions by analyzing communication logs and social network structures within the game. This will help us assess the correlation between these interactions, individual gaming behaviors, and emotional states.

## Data Availability

The raw data supporting the conclusions of this article will be made available by the authors, without undue reservation.

## Footnotes

^1^ https://www.interaction-design.org/literature/topics/user-centered-design

^2^ https://fpesports.pt/

^3^ https://www.counter-strike.net/

^4^ https://store.steampowered.com/

^5^ https://help.steampowered.com/en/- accountdata/SteamLoginHistory

^6^ https://www.faceit.com/

^7^ https://developers.faceit.com/docs/tools/data-api

^8^ https://www.twitch.tv/

^9^ https://pypi.org/project/deepface/
