# Corrigendum: Towards an automated approach for understanding problematic gaming

**DOI:** 10.3389/fspor.2024.1502895

**Published:** 2024-10-14

**Authors:** Ana Paula Afonso, Manuel J. Fonseca, Joana Cardoso, Beltrán Vázquez

**Affiliations:** ^1^ LASIGE, Faculdade de Ciências, Universidade de Lisboa, Lisboa, Portugal; ^2^ Department of Social and Behavioural Sciences, University of Maia, Maia, Portugal; ^3^ Center for Psychology at the University of Porto, Porto, Portugal; ^4^ School of Health, Center for Rehabilitation Research, Polytechnic Institute of Porto, Porto, Portugal; ^5^ Escola Superior de Saúde, Instituto Politécnico do Porto, Porto, Portugal

**Keywords:** video games, Internet Gaming Disorder, gamer behavior, telemetry data, emotional states

A Corrigendum on Towards an automated approach for understanding problematic gaming By Afonso AP, Fonseca MJ, Cardoso J, Vázquez B (2024). Front. Sports Act. Living. 6:1407848. doi: 10.3389/fspor.2024.1407848

**Incorrect Author Name**

In the published article, an author's name was incorrectly written as [Beltran Vasquez]. The correct spelling is [Beltrán Vázquez].

The authors apologize for this error and state that this does not change the scientific conclusions of the article in any way. The original article has been updated.

**Missing Author Contributions**

In the published article, the contributions of Beltrán Vázquez are: [BV: Data curation, Formal analysis, Validation, Visualization, Writing – review & editing], and the correct contribution of Beltrán Vázquez is below:

[BV: Data curation, Formal analysis, Validation, Visualization, Writing – original draft].

The authors apologize for this error and state that this does not change the scientific conclusions of the article in any way. The original article has been updated.

